# *“Green Enough Ain’t Good Enough:”* Public Perceptions and Emotions Related to Green Infrastructure in Environmental Justice Communities

**DOI:** 10.3390/ijerph19031448

**Published:** 2022-01-27

**Authors:** Mahbubur Meenar, Megan Heckert, Deepti Adlakha

**Affiliations:** 1 Department of Geography, Planning and Sustainability, School of Earth and Environment, Rowan University, Glassboro, NJ 08028, USA; 2 Department of Geography and Planning, West Chester University, West Chester, PA 19383, USA; mheckert@wcupa.edu; 3 Department of Landscape Architecture and Environmental Planning, Natural Learning Initiative, College of Design, North Carolina State University, Raleigh, NC 27607, USA; deepti_adlakha@ncsu.edu

**Keywords:** biophilic urban planning, green stormwater infrastructure, social benefits, health equity, emotions, perceptions, mental health, Camden

## Abstract

The concept of biophilic urban planning has inspired neighborhood greening projects in many older urban communities in the USA and beyond. The strengths (e.g., environmental management, biodiversity, heat island mitigation) and challenges (e.g., greenwashing, green gentrification) of such projects are well-documented. Additional research on the relationship between these projects and various social factors (e.g., public perceptions, feelings, and mental health and well-being) is necessary to better understand how people adapt to said projects while struggling to navigate other more pressing socioeconomic issues, especially in communities facing environmental injustice and health inequity. In this article, we focus on one aspect of biophilic urban planning—green stormwater infrastructure (GSI) (e.g., rain gardens, bio-swales, pervious pavements, and wildflower meadows)—in Waterfront South, a post-industrial neighborhood in Camden, NJ, USA, where residents have faced environmental injustices for decades. Our qualitative analysis of in-depth semi-structured interviews of sixteen residents offered a thorough insight into their perceptions and emotions regarding different types of urban GSI projects. Residents acknowledge the many benefits that GSI offers to combat the neighborhood’s social and environmental injustices, but they are cautious about the possibility of some projects prompting new issues and concerns within the community. Our findings reveal potential implications in GSI planning, research, and practice in this neighborhood and similar urban places elsewhere that have yet to undergo gentrification.

## 1. Introduction

The interrelated concepts of biophilia, biophilic design, and biophilic urbanism represent human beings’ affiliation with nature. The need for contact with nature or “biophilia” can be achieved through design approaches in the built environment (biophilic design) and by the systematic integration of nature in cities (biophilic urbanism). While the use of green infrastructure (GI) in urban design is not a new phenomenon, recent years have seen a renewed focus on its implementation around the world. Studies from the USA [1,2], Europe [3,4], Asia [5,6], and Africa [7,8] highlight the wide interest in GI across geographic regions. Although the programs described in these studies rarely use the specific language of biophilic urbanism, its principles are present in the authors’ recognition of GI’s combined environmental, social, and economic benefits.

Numerous studies have highlighted the benefits of biophilic design on human health and well-being, linked to three overarching mind–body systems—physiological, psychological, and cognitive health [9]. Connections with nature can aid physiological responses such as stress reduction, blood pressure decrease, and improved muscle relaxation [9]. Direct physical health benefits include increased longevity and self-reported health, as well as decreased patient recovery times and less need for analgesia [10]. In addition, biophilic design also improves the attention spans, creativity, and productivity of those dwelling in or using spaces designed with biophilic elements. Workplaces that include experiences with nature report higher productivity in their employees [11], in large part because those spaces provide employees with greater opportunities for mental restoration by reducing mental fatigue and boosting brain function [12].

Despite the wide-ranging benefits of biophilia, inequitable distribution of parks and other green spaces continues to exacerbate health inequities across many urban areas [13]. Environmental justice (EJ) research highlights how social privilege enables many communities to enjoy greater access to amenities such as public parks and green spaces. However, EJ communities with low-income, minority, and other socially disadvantaged populations continue to be disproportionately affected with unequal access to natural resources, unbalanced land use practices, and exposure to environmental hazards such as landfills and toxic-emitting facilities. For example, a study on park use in Los Angeles, CA demonstrated how the location and design of parks failed to meet the needs of vulnerable urban communities [14,15]. Studies in Milwaukee, WI, Philadelphia, PA, and Tampa, FL have found that neighborhoods with higher proportions of low-income residents, immigrants, and communities of color have fewer street trees than wealthier ones, making them more vulnerable to the effects of extreme heat and heavy rains [16].

Another line of research underscores how the biophilic planning process has failed to account for long-term, unintended consequences of community greening in many places, especially disadvantaged and EJ communities, in the form of green gentrification [17,18,19,20,21]. While studies have shown that community greening efforts in lower income and minority neighborhoods do not automatically promote health equity [18], they are nonetheless connected to other social factors such as community revitalization, affordable housing, neighborhood walkability, food security, job creation, and youth engagement [22]. Urban green spaces contribute to the cultural and social dimensions of cities, but they are also inherently political; the trend of “greensplaining” can be used as further justification for White privilege, racialized marginalization, and processes of gentrification [23].

Within this context, biophilic urbanists and environmental planners, designers, and engineers globally are incorporating green stormwater infrastructure (GSI) in the form of rain gardens, bio-swales, biodiverse plantings, wildflower meadows, permeable paving, tree trenches, stormwater planters, rain barrels, vegetated roofs, and other landscape design elements [24,25,26]. This widespread incorporation is due in large part to researchers’ identification of GSI as a promising approach to help low-income communities adapt to climate change through the provision of multiple ecosystem services [27]. GSI projects help to conserve, restore, or enhance natural areas; address environmental concerns related to stormwater runoff, flooding, and combined sewer overflow events; and provide additional ecological benefits such as wildlife habitats and temperature moderation [28,29,30]. These projects can also improve the aesthetic quality of neighborhood streets, sidewalks, bicycle lanes, and public spaces by making them pleasant and safe for walking, cycling, and active commuting [31]. 

Despite increasing interest in GSI, significant barriers remain for its widespread implementation. Ironically, the wide support of GSI can also work against it. As government programs tend to be highly specialized and siloed, many agencies may recognize the benefits of GSI without necessarily taking ownership of its implementation, as the process may fall within the purview of multiple agencies [32]. In a study of GSI implementation in Portland, OR, Thorne et al. found that sociopolitical barriers (e.g., concerns over long-term support and public preferences) were more important than technical or scientific barriers [33]. Indeed, while policymakers are increasingly adopting these programs, much less is known about the public perceptions of and values relative to GI, especially GSI, or the willingness of private property owners to either support neighborhood programs or install GSI on their own properties, both of which are crucial for successful implementation [34,35]. 

While much of the research on the benefits of GSI has drawn on prior studies on green spaces and vegetation more broadly, GSI refers to a wide range of practices, which raises some questions as to the applicability of all green space benefits to GSI. In translating generic GI or green space research to GSI, the literature highlights three interconnected concerns when it comes to public perception and the implementation of GSI: knowledge, values, and context. Knowledge refers to the awareness of the benefits of GSI, values to the extent to which residents or GSI users value those benefits, and context to the extent to which the benefits themselves or their assigned values vary based on the specific neighborhood in which the projects are implemented or proposed.

Because GSI is inherently diffuse in comparison to traditional “grey” stormwater infrastructure, it relies on a wide array of stakeholders, especially private property owners, to be implemented at scale. However, increasing awareness of the benefits of GSI among practitioners has not necessarily translated into awareness or interest in those benefits among the public. For example, a study in Rotterdam, the Netherlands, showed that while GI was being implemented as part of a long-term climate change strategy, residents did not necessarily perceive a direct connection between GI and climate mitigation [36]. In general, the survey showed that residents were much more cognizant of direct benefits such as recreation and aesthetics than indirect benefits such as temperature reduction and carbon storage. While this study focused more generically on GI rather than on GSI specifically, this perception is likely to be more pronounced for GSI, which is both more varied and less well understood by the public. While neighborhood residents may not have the ecological or engineering knowledge of practitioners, there is also a knowledge gap in the other direction, where residents have greater experiential knowledge of their communities and how GI directly affects them [37]. This gap becomes even more relevant when considerations move from knowledge alone toward incorporating both values and context. 

While GSI is used as an umbrella term for a range of practices, it is unreasonable to assume that all forms of GSI provide all the benefits ascribed to GSI more broadly. There are considerable variations, for example, in how aesthetically pleasing residents find GSI, even when they agree that all forms of it are improvements over non-vegetated landscapes [38]. Further complicating the relationship between GSI and understanding its benefits is how several of the practices included within broad GSI programs in the USA do not necessarily involve vegetation. Philadelphia’s GSI program, for example, includes impervious pavement and rain barrels within its practices [39].

Related to these concerns regarding the understanding of the benefits of GSI are questions as to how residents value these benefits. The mere fact that a benefit exists does not guarantee that people will care about or be willing to invest time or money to support it [40], especially in the context of specific neighborhoods [35]. While some residents may perceive GSI as beneficial in theory, others may express concern that land devoted to GSI will be taken from affordable housing, thereby suggesting important tradeoffs that studies about residents’ perceptions of GSI in general may not reflect. It is crucial, therefore, for researchers to incorporate into their studies in-depth interviews with residents to better understand their perceptions of GSI within specific neighborhood contexts, as well as to privilege this local knowledge over the researchers’ own observations of GSI projects in those same contexts [41].

In summary, the current research has solidly established the environmental, social, and economic benefits associated with well-known aspects of urban nature, GI, and various types of nature experiences. Few studies, however, have explored how GSI affects human health while providing other social and economic benefits, and even fewer studies have explored the extent to which GSI projects impact human health, especially mental and emotional health and well-being in EJ communities. Although the studies of residents’ perceptions of GSI remain scarce, they nonetheless highlight the importance of understanding local contexts and recognizing that practitioner or designer perceptions of what is best for a community may not align with the perceptions of the people who actually live there [41]. These lived experiences and local perceptions are directly tied to the concept of place attachment, which refers to the cognitive or emotional bond that may form between a person and a place [42]. The stronger a person’s place attachment, the stronger their quality of life and tendency to care for the place will be [43,44,45,46], as well as their ability to develop more constructive or adaptive behavioral strategies [47]. Many questions still exist, however, regarding the range of concerns that residents might have about GSI projects, how those concerns vary based on the types of GSI at hand or the specific EJ community context, and the best ways to solicit and incorporate the perceptions and feelings of those residents into GSI planning and design.

Based on our literature review above, the primary aim of our study is to understand urban residents’ perceptions of and concerns regarding various GSI projects and their potential benefits, how the design and context of those GSI projects influence residents’ perceptions, and how these projects can enhance the efforts of EJ communities toward achieving health equity. The following three research questions capture our study’s aims:

(1) How do urban residents perceive the social benefits and potential challenges of, as well as their emotional attachments to, various types of GSI projects?
(2) How do the physical design characteristics and placement contexts of GSI projects enhance residents’ perceptions?
(3) To what extent can GSI and other community greening projects support EJ community and health equity efforts and initiatives?

## 2. Materials and Methods

### 2.1. Geographic Context

**Figure 1:**
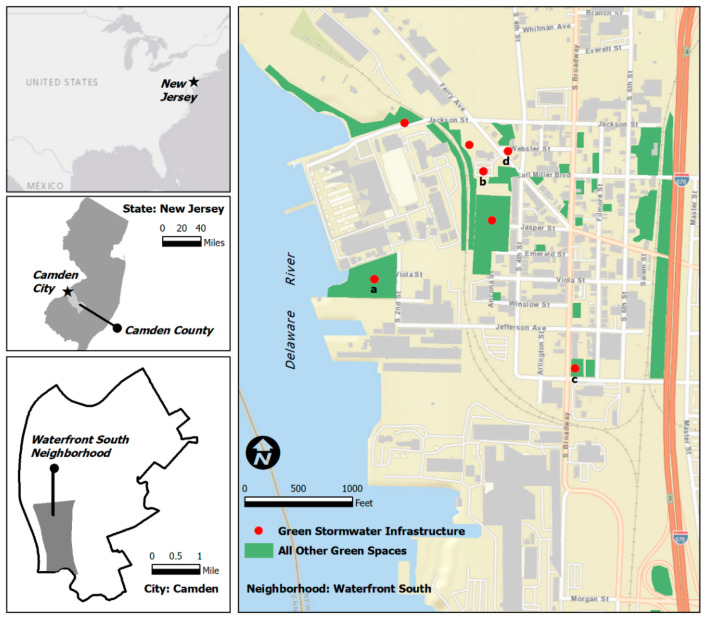
Map of Waterfront South, its existing GSI projects, and other formal or informal green spaces (photos of the four marked projects above (a–d) are detailed in Figure 2).

The research questions of this study motivated us to focus on the Waterfront South neighborhood (Figure 1) within the City of Camden in New Jersey, which is an EJ community with noticeable recent “greening” efforts. This post-industrial or “shrinking” neighborhood, which was added to the U.S. National Register of Historic Places in 1990, is in the southern portion of Camden and shares its western edge with the Delaware River. Table 1 presents sample demographic data based on the five-year estimates of the U.S. Census Bureau’s American Community Survey [48].

Residents of Waterfront South have been facing various forms of environmental injustice for many decades. Following the collapse of its largest employer, the New York Shipbuilding Corporation, after World War II, suburbanization, disinvestment, and population decline decimated the neighborhood. The community was repeatedly targeted for industrial use, along with municipal and county facilities, thereby exacerbating the residents’ pollution-related health issues. This small neighborhood is currently home to the Camden County Municipal Utilities Authority, a county-wide wastewater treatment plant, as well as an incinerator that serves all of Camden County’s 37 municipalities. Moreover, an assortment of port and warehouse facilities, licorice and gypsum production plants, scrapyards, and other light industrial uses generate heavy truck traffic day and night. These industries and plants create a toxic mix for the residents, causing cancer, asthma, and other illnesses; occasionally intolerable strong odors; and soil, air, and water pollution [49]. The City of Camden has a combined sewer service (CSS), which frequently overflows due to higher rates and frequency of precipitation, causing dangerous blackwater overflows that pollute the Delaware River and other nearby waterbodies, as well as streets and even residents’ basements. Notable social issues in this neighborhood include high poverty and crime rates, including illegal activities such as drug trafficking and prostitution. According to a regional equity mapping study, almost all parts of Camden, including Waterfront South, are potentially disadvantaged because of the combination of elements described above [50].

**Figure 2:**
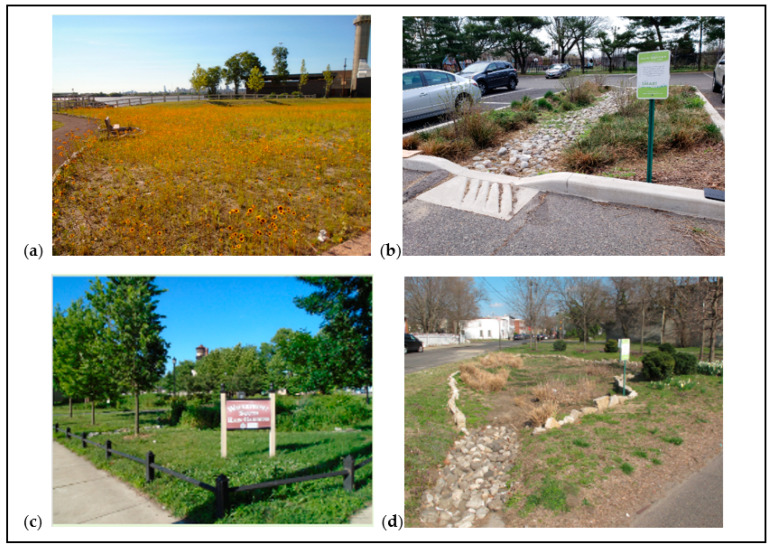
Photos of sample GSI projects in Waterfront South (specific locations marked on map in Figure 1): (**a**) a wildflower meadow in Phoenix Park; (**b**) a small rain garden in a publicly owned parking lot; (**c**) a pocket park comprising several rain gardens and vegetated areas; (**d**) a rain garden in a publicly owned vacant lot. All photos were captured by the lead author, Summer 2019.

The neighborhood, however, has “enough” community greening projects compared with many other EJ communities of similar size and context. There are two parks, five pocket parks, multiple strips of wooded areas next to highway or railway corridors, and several city-owned or -managed vacant land parcels. To address stormwater and flooding issues and impervious vacant lots, government agencies and non-profit partners have constructed many GSI projects in recent years, including rain gardens, porous concrete surfaces, shade trees, and wildflower meadows, to meet federal requirements and minimize combined sewer overflows (CSOs) (Figure 1 and Figure 2).

### 2.2. Selection of Participants

This article is based on qualitative data collected as part of a broader project titled “Greening Camden Waterfront South”, conducted and led by the primary author between 2018 and 2020 [49], the purpose of which was to create a green stormwater infrastructure plan for the neighborhood. The project team partnered with two community-based organizations and engaged community residents in several ways, including an initial public meeting and workshop, photovoice activities, neighborhood observations, and focus groups. The partnering organizations, Heart of Camden and Camden Fireworks, recruited participants through local announcements and door-to-door outreach. At the initial public meeting and workshop event, which attracted about 40 stakeholders, the project team introduced the purpose, meaning, and needs of GSI planning in the neighborhood and recruited eighteen people who expressed initial interest in participating in the follow-up activities. Through the information presented in these engagement sessions and activities, participants gained a basic knowledge about the need for and benefits of GSI projects in urban neighborhoods such as Waterfront South. At the end of the engagement activities, those eighteen stakeholders were invited to participate in face-to-face interviews and were offered compensation in the form of gift cards for their time. Out of the original eighteen participants, sixteen agreed to the interviews, who are the focus of this article.

Of the sixteen participants, eight were lifelong residents of Waterfront South, while the rest had lived there between 5–20 years. About 72% of participants were female and 28% were male, with ages ranging from late 20s to late 80s. About 40% were Black, 33% were Hispanic or Latinx, and 27% were White. The participants’ median household income was close to the neighborhood median of $23,520, although this was the only aspect where the demographic composition of our participants mirrored the neighborhood average. Finally, nine participants had full- or part-time jobs at schools or non-profit organizations, while the remaining participants were homemakers, retirees, or unemployed.

### 2.3. Data Collection

The primary author and a research assistant interviewed thirteen participants on three separate days in August 2019 at a community meeting space inside the main office of a local non-profit organization. Additionally, one participant was interviewed at their home and two others by phone. Each interview lasted approximately one hour, and all were recorded with permission from the participants. The project team conducted semi-structured interviews so questions could be modified for participants of different backgrounds and age groups, while maintaining the central meaning of each prompt [51]. The interviewers followed a set of fixed questions that occasionally provided photo or map prompts to elicit specific information, which also occasionally evoked open-ended conversations. The team developed these questions in close consultation with the partnering organizations to encourage participants to think critically about their attachments to GSI projects and their overall community.

Each interview began with general questions about participants’ perceptions of different types of GSI, where interviewers displayed printed maps and images of GSI projects from the neighborhood and beyond for reference (e.g., Figure 1 and Figure 2 above). Next, the interviewees answered questions related to the perceived benefits of GSI, as well as their perceptions of and emotions connected to their community’s GSI projects. Finally, participants answered questions asking them to explain the issues that GSI projects generate for the neighborhood, the challenges related to the GSI planning and design processes, and potential ways to overcome those challenges.

In addition to these interviews, the project team conducted field observations of the study area to collect notes on GSI project locations and their characteristics, such as appearance, signage, and visibility. These field observation notes were used to ground-truth interview findings and gain a better understanding of the participants’ perceptions of specific GSI projects. 

### 2.4. Data Analysis

All interview recordings were transcribed by a professional service. The primary author and two research assistants reviewed the resulting transcripts and confirmed any logistical information contained therein (e.g., location of a particular GSI project, types of GSI, land ownership, amenities present at the project sites), and compared such information with notes taken during the field observations. In a few cases, the team updated incorrect information. For example, one interviewee commented on Phoenix Park and its amenities but referred to it as Liney Ditch Park. Another interviewee criticized the design of a rain garden but mentioned “planters”. Another one reported missing signage in one of the rain gardens, but the team discovered visible signage during the field observation. The main content of the interviews, such as people’s opinions or feelings about GSI, however, was not edited. 

Next, the team conducted a qualitative content analysis and coded all transcripts using the NVivo qualitative analysis software. The team used standard qualitative coding procedures, starting with open coding and progressing through axial and selective coding as new concepts emerged [52,53]. Initial codes were created following interview questions. While examining the transcripts, the team discussed emergent ideas expressed by the interviewees and recoded the transcripts based on these new ideas [54], which eventually became the core of this analysis. Table 2 describes open codes and their properties related to the research questions, while Table 3 shows axial codes and the selective code based on open codes. Finally, for the open code “GSI emotions”, the team further categorized emotions expressed by the participants according to Plutchik’s emotion wheel’s middle ring, which comprised eight basic emotions organized in contrasting pairs: joy and sadness, anger and fear, trust and disgust, and surprise and anticipation [55].

## 3. Results and Discussion

### 3.1. GSI Social Benefits, Concerns, and Emotional Attachments

In response to our first research question, we found that urban residents in EJ communities perceive GSI through its social benefits, social concerns, and emotional attachments.

Perceived social benefits of GSI: The interviewees generally agreed that most GSI projects could offer social and environmental benefits as long as they were properly designed and maintained and did not generate additional concerns or nuisances for the community. Several participants thought the social benefits would factor in more highly than environmental benefits would (e.g., water quality improvement and flood mitigation). One participant said, “I put ecology at the forefront of my thinking and I consider GSI serving ecological benefits. That’s not necessarily what my neighbors might be thinking though. [They are thinking about] flooding in their basements due to heavy rain. So, GSI serves people wherever it impacts them”. Another participant who advocated for more tree trenches said, “Everyone knows how trees are important to improve air quality in polluted industrial neighborhoods like ours, but they appreciate trees more because trees give shade in summer months, take up lots of water so that your basement doesn’t flood, and some trees have beautiful flowers and colors in spring and fall”. Conversely, most of our participants were not convinced about the potential economic gain from neighborhood-wide GSI projects. Only one person, who had to cleanup blackwater from basement flooding caused by heavy rains, anticipated that more GSI projects, if strategically located and properly functioning, could eventually protect residents from financial loss.

Many participants valued the social benefits of GSI the most because they considered GSI as part of community green space or landscape inventory that warrants benefits like other green spaces do. These participants’ interests were, therefore, limited to visible projects such as rain gardens and bio-swales that included perennial plants and flowerbeds. According to most participants, GSI could increase neighborhood appearance through beautification. One participant said, “Plants and flowers in the rain gardens or bio-swales help me breathing, but the main reason I like them is their beauty and scent”. Another participant added, “I love seeing [rain gardens] that have flowers… beautiful. Generally, I love seeing green growing things in the urban environment. I think we need it for our mental health … for our souls”. Relatedly, another participant remarked, “I am not sure how much water stormwater planters can manage but they dress up your porch or front yard if you have one”. Indeed, another participant agreed by stating, “[GSI projects] are contributing something back aesthetically to the area… and bringing value back... Any green areas of gathering as [a]…place for residents and wildlife”. For these reasons, a large portion of residents responded positively to GSI only in early spring when flowers blooms or plants are in early stage of growth but not in summer when plants grow too long or too dense or winter when plants die, making the projects look similar to dead spaces or “dumping grounds”.

Perceived social concerns of GSI: Participants’ perceptions of GSI-related social concerns were connected to maintenance issues at both community and individual levels. At the community level, the GSI maintenance issue was the biggest concern among participants. In particular, GSI projects, if not properly or regularly maintained or monitored, could become a nuisance to the community due to trash accumulating within the project area. One participant said, “[GSI projects] have to be truly functional, otherwise they are a nuisance to me”. For any type of GSI, “the weeds always come up in between and when they’re not maintained or kept properly, they begin to look like an eyesore. From early growing season to fall or winter—they go from beautiful to ugly. The accumulation of trash makes it worse”. Many people highlighted a connection between “trash and negative uses”, with vandalism in GSI project areas being the most common concern. As one participant recalled, “We planted some flower beds [in a rain garden] but someone vandalized them”.

A few participants pointed out that maintenance workers sometimes mistakenly mow areas with perennial plants, rationalizing their concerns as “a matter of respect for” and a “lack of knowledge” on behalf of the maintenance crews. Some participants advocated for community-initiated cleanup activities rather than relying on the city contractors who only clean up once or twice a year, although safety remains at the forefront of their minds. Indeed, as one participant described, “[It is] not easy to clean up trash from [neighborhood GSI and parks] even if we want to. You must need a pair of gloves because you can find needles in them. I am a teacher and want to take my kids to do some clean up in this neighborhood but the safety or needles question always is a barrier. It would be better if we can also engage the parents for 10 min per week”.

At the individual level, participants had polarizing opinions about rain barrels. Some had one or more rain barrels in their front and backyards (if available) and they made good use of those barrels for their small gardens. Participants who did not like them (even if they obtained them at no cost from the city government or non-profit agencies) did not know where to place them in their limited spaces, did not have time to maintain them, or felt that rain barrels were ugly (even if organizations in the neighborhood hosted creative workshops to paint the barrels). Therefore, they felt that rain barrels were a waste of time and space and did not offer any benefits. 

Emotional attachments and responses to GSI: All participants agreed that any type of green space or project should generate positive emotions. In a neighborhood largely characterized by industrial waste, illegal dumping, and widespread trash accumulation, a nicely maintained green space, even if it is a small rain garden, could “make you happy on a rough day”. Many participants, however, commented that the same projects could trigger negative emotions if they did not function as intended or caused other issues. Table 4 is a compilation of sample quotes representing interviewees’ different emotions. 

In summary, residents demonstrated positive perceptions and emotional or place attachments with GSI projects if they added visual appeal, functioned as intended, and were maintained regularly. Our participants acknowledged the physiological value and health benefits of GSI projects in the same way as participants in other studies recognized the broader benefits of biophilic design concepts [9,12]. Our findings also aligned with those of other studies demonstrating that public perceptions of or preference for GSI projects more heavily relied on direct benefits (e.g., appearance, visual appeal, recreation) than indirect benefits (e.g., carbon sequestration, heat island mitigation) [36]. Our participants expressed a greater number of positive attachments to GSI projects with vegetation (e.g., rain gardens) but reported that the same projects could provoke negative emotions in light of improper maintenance, again consistent with prior research [38,56].

Some negative perceptions related to the social benefits of or concerns regarding GSI stemmed from a lack of public knowledge about or perspectives of GSI or stormwater governance, which is consistent with prior research [57]. Other studies have demonstrated that residents’ preferences and understanding of GSI governance was crucial for successful implementation, so that residents could feel ownership of GSI projects [32,34,35]. Many of our participants, while excited about bringing nature into their community, were not consciously aware of biophilic design and its social and ecological benefits, other than its positive physical appearance, its ability to generate strong place attachments, and its power to convert people’s emotional attachment into a greater quality of life; prior research has documented the importance of these factors [42,46,58,59].

One potential solution for overcoming the lack of community environmental education or engagement is through a careful design of decision-making and maintenance-planning processes prior to undertaking GSI installations. Participatory planning tools should be incorporated early and often to create positive public perceptions of GSI, encourage community ownership of the projects upon construction, and increase the likelihood that residents will perceive projects as community assets. Residents can get involved in the selection of a site (e.g., sidewalk vs. parking spot), the type of GSI project (e.g., rain garden vs naturalized area), or the type of plant palette (e.g., ornamental grass vs. wildflowers) by participating in early-stage design workshops [60]. These interpretations and recommendations align with prior research highlighting the importance of participatory planning processes in improving community acceptance, investment, and interest in GSI installations [61,62,63].

### 3.2. GSI Design and Placement Context

In response to our second research question, we found that urban residents perceive GSI based on its design and placement context. Using creative, adaptive, and customized GSI designs can increase the social benefits of GSI.

Most participants felt that GSI design should be sensitive to and appropriate for the neighborhood context. No one appreciated a “one design fits all” or “cookie-cutter design” approach. For example, stones or rocks are commonly used as design materials in many GSI projects (e.g., rain gardens, bio-swales), but they can be used for vandalism in some neighborhoods and would need to be avoided accordingly. With this neighborhood-specific knowledge in mind, a small number of participants stressed the designer’s role in the creation and implementation of GSI projects. One participant asserted that “[GSI] planning must be done by professionals”. They also emphasized the importance of signage and visuals as community environmental education tools to inform people about the purpose that GSI projects serve and how they should look in all four seasons.

In light of participants’ endorsements of context-sensitive design, many believed that GSI projects with a dual purpose (e.g., ones that blend environmental and social aspects) would better serve the neighborhood’s environmental goals. One participant even offered some design options: “You leave a low-elevation grassy area for kids to play and make it a shallow pond when it rains… You have a bus shelter and put a green roof on top. You connect a series of tree trenches with seating arrangements for community residents as hangout [spaces]”. Another participant added, “A park is known for bringing people together but [GSI] is like a walk by. But we can still add some park-like features if the project is big and there is room”. Participants generally preferred GSI projects when combined with other placemaking features such as benches, small sculptures or other art installations, solar-powered lamp posts, or aesthetically appealing and visually obvious signage. 

City residents who grew up in the suburbs demonstrated a higher appreciation of GSI projects or green spaces in general. Many participants thought flow-through planter boxes were aesthetically pleasing and “suitable for a dense urban environment” and could “enhance the neighborhood appearance if maintained”. However, they did not think all types of GSI projects suitable in a suburban setting would be socially accepted or welcomed in urban neighborhoods. For example, the majority of participants were apprehensive about naturalized areas or wildflower meadows. One participant said, “They are more appropriate for suburbs. Here in our neighborhood, no one wants another naturalized area…a fancy name for unobserved, abandoned vacant lots”. “They grow enormously and make the place trashy”, another person explained, “They are okay in the beginning, but when the wildflowers die, they look horrible. It makes a vacant lot look even worse”. Anticipating this kind of negative reaction, another resident echoed the group’s preference for projects with a dual purpose: “A combination of naturalized areas and mowed grassy areas might work better. In that case, people will know the naturalized area is kept like this by design. There should be visible and nicely designed signage and trashcans nearby so that people realize naturalized areas are not for trash”.

Participants also expressed reservations about three additional types of GSI projects: ones with deep underground water storage, stormwater bumpouts, and tree trenches. With the water storage projects, participants worried that constructing them close to their homes might cause basement flooding, which in turn would eclipse any of the projects’ environmental benefits. Some also worried these storage projects would become breeding grounds for mosquitos. Participants were hesitant to embrace bumpouts because of the possibility of losing coveted parking spots along their neighborhood roads. Finally, some participants were worried about tree trenches resulting in bird droppings on cars if they were located close to parking spots or their root systems causing pipes to break if they were located close to terracotta drainage pipes.

Based on interviewees’ responses related to our second research question, our findings are conceptually consistent with prior research done by Travaline et al. [35], but our participants also offered many new insights. We argue that creativity can and should play a major role in designing GSI site plans [60] and choosing plant palettes [64]. If, for example, the only low-cost solution for a community is to convert vacant lots into naturalized areas, then designers could create a buffer of a regularly mowed grassy area between the sidewalk and the naturalized area. That strip of grass, along with appropriate signage and a simple fence, may help people understand the intentional design approach of the naturalized area. In terms of a creative plant palette, designers should choose a variety of plants that will provide a pleasing array of colors and textures in the spring, summer, and fall seasons. Careful design considerations can be explained by using tree trenches as an example. Before constructing tree trenches, it is important to understand the width and conditions of surrounding sidewalks, as well as the locations of underground pipes or wires that connect properties, so that projects do not damage existing infrastructure or cause any issues for pedestrians. Creative design considerations should also offer dual purposes or combine multiple uses (e.g., blending stormwater management, play spaces, and community educational spaces) to encourage direct public interactions with the GSI projects [41].

### 3.3. GSI, EJ, and Health Equity

In response to our final research question, we found that urban residents perceive that community greening alone does not guarantee EJ and health equity.

All participants felt strongly about the environmental injustices they and their community have faced over the decades and currently continue to face. Industrial land use is predominant in this neighborhood, and most participants mentioned strong odors coming from the wastewater treatment plant. As one participant said, “a small neighborhood of 1000 residents has to bear the load of more than 500,000 people”. Another resident echoed this concern, adding that, “It’s not only the odor issue, there is a consistent issue with poor air quality as industries such as a gypsum plant and an incinerator release toxic air”. These issues are “on top of what the residents are already facing from unmanaged vacant lands, trash piling, heavy truck traffic, illegal dumping, drug trafficking, and prostitution”, according to the same participant. Many participants labelled these issues as related to both EJ and health equity, some believing that community greening projects only partially address these issues, despite their benevolent intentions of wanting to give back to the community. Residents were grateful, however, that green gentrification [17,18], which is currently occurring in many neighborhoods throughout the city of Philadelphia across the Delaware River, has not yet affected Camden. 

One possible explanation for residents’ reluctance to see GSI projects as the answer lies in the reality that environmental injustices in this community are “so significant” and warrant greater interventions. A small number of participants argued that Waterfront South will see justice only when industrial pollution fully stops, local residents are offered employment by these industries, greening projects are well-maintained, and projects are safe and secure for people and children of all ages to use. Many interviewees were equally reluctant to accept the benefits of planning outright, with one saying that “planning is good, as long as [ideas] get materialized” and does not change the “look and feel” of the neighborhood (i.e., resists green gentrification). Another participant emphasized the need for community engagement, valuable only if practitioners “put our suggestions in the plan, and somehow engage [residents] in the implementation process”.

All participants generally agreed that, unless all these criteria were satisfied and GSI projects were connected to initiatives that address larger socioeconomic issues such as unemployment, insufficient or subpar housing, and crime—“Green enough ain’t good enough”. Additionally, participants agreed their neighborhood contained “more than enough” green spaces and projects, thereby offering a stark contrast to prior researchers’ findings that disadvantaged urban communities typically experience an inequitable distribution of GI [13,65]. For Waterfront South residents, the issues with GSI lie in the design, amenities, and improper maintenance of existing projects, along with the co-existence of toxin-emitting facilities, prolonged exposure to environmental hazards, and the failure of local government to meet residents’ needs—harsh realities observed in multiple previous studies in other similar communities [14,15,16].

## 4. Conclusions

In this article, we have presented residents’ perceptions of and emotions attached to biophilic urban planning and the neighborhood greening process, with a distinct focus on GSI planning, in a post-industrial and historic neighborhood that has faced environmental injustices for decades. Our findings suggest that urban residents perceive GSI projects in three major ways:

*Perception 1:* GSI perceptions are captured through the discussion of the social benefits of and concerns about GSI. Residents acknowledge the social and environmental benefits of GSI, but they value social benefits more because they believe the social benefits are more visible and the experience is more direct. At the same time, improper or infrequent maintenance can not only minimize or even diminish the environmental benefits but also disqualify the social benefits. When focusing on different types of GSI, residents feel that not all GSI projects are socially accepted in urban neighborhoods, particularly naturalized areas, wildflower meadows, and tree trenches. More community environmental education is needed so residents can be more aware of the purposes and functions of GSI and assist with their maintenance. For all of these reasons, GSI projects can trigger both positive and negative emotions.

*Perception 2:* GSI projects are perceived through their design and placement context. Residents believe that creative, adaptable, and customized GSI design that is sensitive to the needs and context of a neighborhood can increase social benefits. Planners and policymakers must, thus, resist a “one size fits most” approach when it comes to designing GSI projects for EJ communities.

*Perception 3:* Residents in disadvantaged urban neighborhoods perceive GSI using the lens of EJ and health equity. GSI projects, as part of broader community greening initiatives, do not automatically guarantee EJ and health equity, which may be absent in many shrinking cities. Rather, these projects may appear in the form of greenwashing, causing green gentrification and other forms of potentially unintended social problems.

Our findings are important for facilitating a greater understanding of the connection between biophilic urban planning or urban greening with EJ and health equity. Research on this topic, especially the connection between GSI and EJ or health equity, is emerging but inadequate. This study focused on people’s perceptions and emotions because those are important aspects of mental health and well-being. The COVID-19 pandemic has reminded us of the importance of having access to green infrastructure, its restorative effects [66], and the positive correlations among views of and access to green infrastructure and physical and mental wellness [66,67,68]. Our research suggests, however, that merely having physical access to green spaces and greening projects is not enough to ensure people’s well-being. Our participants agreed that they had access to “enough” greening projects, but they do not experience the full benefits of that easy access due to various contextual factors: issues of safety and security, design and maintenance, urban wildlife management, inadequate educational signage, and professionals and practitioners not understanding the true needs of the community. Our participants, therefore, did not feel that GSI and other greening projects necessarily addressed EJ and health equity, even if the projects were well-intentioned. These findings are consistent with prior research on the social benefits of GSI in other cities [41,64,69,70].

Residents will most likely not appreciate designers simply implementing more greening projects in EJ communities, often by converting vacant and hazardous lands into GSI projects, without honest public engagement and without considering the context, outcomes, and an achievable maintenance plan, because “green enough ain’t good enough”. Therefore, we argue that public engagement should be integral to the GSI planning, construction, and maintenance processes to increase the social benefits of GSI and better facilitate people’s adaptation to biophilic urban planning or urban greening projects. We suggest that GSI and biophilic urban planning professionals should meaningfully engage the communities by listening to local residents, valuing their local knowledge and experience, and understanding their perceptions of and emotions related to such projects so they do not introduce new social issues in the neighborhood. Urban greening, even if done in consultation with local residents, should coordinate with other projects addressing larger socioeconomic needs of the communities.

The findings from this study may be applicable to similar neighborhoods with a history of environmental injustices and neighborhoods that have not yet experienced or are at risk of soon experiencing gentrification. While these findings are important, we acknowledge the limitations of our study. Our findings and conclusions are based on qualitative data and analysis. The sample size (n = 16), while appropriate for a qualitative study, was small, so the results should be interpreted with caution. Further research using larger data samples and more participants is needed before we can fully quantify the relationship between the social benefits of GSI and community perceptions. This study was conducted among residents of one neighborhood in Camden, thereby restricting the generalizability of our findings beyond the study area. The in-depth, semi-structured interviews, however, provided rich, qualitative data on people’s perceptions, feelings, and emotions about GSI planning or biophilic urban planning more broadly. Overall, our findings highlight that GSI projects can be cost-effective and resilient approaches that can generate many social, economic, public health, and environmental benefits for communities, although extreme caution needs to be taken during the planning, implementation, and maintenance phases so that these projects can not only address environmental issues but also serve the communities socially. Future studies focusing on this line of inquiry can compare social perceptions of GSI in multiple cities from different regions of the USA or different parts of the world or explore differences in these perceptions between cities and suburbs. Future studies can also focus on other forms of biophilic urban planning projects by following a similar methodology and comparing findings regarding GSI projects and other types of community greening projects. 

In conclusion, the benefits of GSI are likely to be achieved most successfully if the creation and management of green spaces are integrated with traditional land development, infrastructure, and built environment planning. Integration of GSI with the surrounding built environment is crucial for maximizing the benefits for equitable urban development and public health. The real potential of GSI will be realized only if activities or operations undertaken for its planning are supported by the whole community.

## Figures and Tables

**Table 1:** Sample demographic data for Waterfront South (Census Tract 6018).

Topic	Value	Additional Notes
Population	1082	Total area 0.8 square mile
Median age	35 years	About 62% people are between the age group 18–64
Gender	Male 57% Female 43%	Female population in both Camden City and Camden County is above 50%
Race and Ethnicity (selected)	Black 41% White 6% Hispanic 48%	Black population in Camden City 39%, Camden County 18% White population in Camden City 6%, Camden County 57% Hispanic population in Camden City 51%, Camden County 17%
Median household income	$23,520	Camden City $27,015, Camden County $70,451
Per-capita income	$11,900	Camden City $15,001; Camden County $35,958
Percent of population living in poverty	32%	36.4% in Camden City, 12.2% in Camden County. Notably, 80% of seniors (age 65 and over) in the neighborhood are under the poverty threshold.
Mean travel time to work	32.7 min	Common modes of travel: private vehicle (50%), carpool (31%), public transit (19%).
Number of households	448	About 37% of households are headed by only females compared to 20% by only males. The rest of the households are headed by married couples or non-families.
House vacancy	22%	16% in Camden City, 9% in Camden County
High school diploma	75%	Ages 25 and older

**Table 2:** Open codes emerging from qualitative content analysis of interview transcripts.

	Open Codes	Description
Research Question 1	Understanding of GSI	The concept and function of GSI, various types of GSI projects, urban versus suburban or rural GSI projects, regulatory aspects, and financial aspects.
	GSI social benefits	The social and health (physical or mental) benefits of GSI of all types in urban settings.
	GSI issues	The issues or challenges generated by various urban GSI projects.
	GSI emotions	People’s emotional attachment to various types of urban GSI projects.
Research Question 2	GSI design	Design aspects of urban GSI projects, including landscape design, plant palette, design concepts, and context.
	GSI context	Understanding the role and meaning of GSI in urban landscapes and neighborhood contexts.
	GSI signage and community education	The use of GSI projects as a form of community environmental education through signage, flyers, websites, or workshops.
	GSI planning process	Understanding GSI as part of community greening and biophilic urban planning processes.
Research Question 3	GSI and EJ	The role of GSI in EJ communities, GSI as an intervention technique to combat environmental injustices, GSI as a barrier to EJ.
	GSI and health equity	The role of GSI in addressing issues related to health equity and overall community health.

**Table 3:** Axial codes and selective code based on the open codes.

Open Codes	Axial Codes	Selective Code
Understanding of GSI, GSI social benefits, GSI issues, GSI emotions, GSI and community education	Perception of GSI social benefits, concerns, and emotional attachments	Public perception of GSI
GSI design, GSI context, GSI planning process	GSI perception through its design and placement context	
GSI and EJ, GSI and health equity	GSI perception through the lens of EJ and health	

**Table 4:** Residents’ emotional attachments and responses to different types of GSI.

Type of GSI	Sample Quotes	Emotions
A community park with shade trees	“I feel happy when I go for morning walks in the nearby park. The sight of the trees and flower beds gives me mental comfort.”	Joy
A community park a wildflower meadow	“I was pleasantly surprised to explore Phoenix Park the first time I saw it. The wildflowers were so cool.”	Surprise
A parking lot with porous pavements	“I couldn’t believe it when I heard about the actual function of those parts of the lot. More education is needed, indeed.”	
A rain garden on a parking lot	“Sometimes these projects, and the people and organization who maintain them, help me keep my trust in people and society.”	Trust
A rain garden in a street intersection	“Someone threw plastic bottles over there and blocked the outlet. I was so mad. How can people do that?”	Anger
A rain garden park	“The stones over there scare me. There are some rude kids. You never know what they could do with those stones just for fun. My windows are very close.”	Fear
Multiple types/GSI in general	“I feel sad when I realize the city and other agencies are investing a lot of money to build these projects but there is not enough maintenance. Also, we have so many green projects here but how many are useable? Should we do more greening or focus on other more pressing issues and maintain whatever we’ve got already really well?”	Sadness
